# Parkinson’s Disease and Photobiomodulation: Potential for Treatment

**DOI:** 10.3390/jpm14010112

**Published:** 2024-01-19

**Authors:** Brian Bicknell, Ann Liebert, Geoffrey Herkes

**Affiliations:** 1 NICM Health Research Institute, University of Western Sydney, Westmead 2145, Australia; ann.liebert@sydney.edu.au; 2 Sydney Adventist Hospital, Wahroonga 2076, Australia; 3 Faculty of medicine and Health, Sydney University, Camperdown 2050, Australia; 4 Neurologist, Sydney Adventist Hospital, Wahroonga 2076, Australia; geoffry.herkes@sah.org.au; 5 College of Health and Medicine, Australian National University, Canberra 2600, Australia

**Keywords:** Parkinson’s disease, photobiomodulation, transcranial, remote, neuroprotection

## Abstract

Parkinson’s disease is the second most common neurodegenerative disease and is increasing in incidence. The combination of motor and non-motor symptoms makes this a devastating disease for people with Parkinson’s disease and their care givers. Parkinson’s disease is characterised by mitochondrial dysfunction and neuronal death in the substantia nigra, a reduction in dopamine, accumulation of α-synuclein aggregates and neuroinflammation. The microbiome–gut–brain axis is also important in Parkinson’s disease, involved in the spread of inflammation and aggregated α-synuclein. The mainstay of Parkinson’s disease treatment is dopamine replacement therapy, which can reduce some of the motor signs. There is a need for additional treatment options to supplement available medications. Photobiomodulation (PBM) is a form of light therapy that has been shown to have multiple clinical benefits due to its enhancement of the mitochondrial electron transport chain and the subsequent increase in mitochondrial membrane potential and ATP production. PBM also modulates cellular signalling and has been shown to reduce inflammation. Clinically, PBM has been used for decades to improve wound healing, treat pain, reduce swelling and heal deep tissues. Pre-clinical experiments have indicated that PBM has the potential to improve the clinical signs of Parkinson’s disease and to provide neuroprotection. This effect is seen whether the PBM is directed to the head of the animal or to other parts of the body (remotely). A small number of clinical trials has given weight to the possibility that using PBM can improve both motor and non-motor clinical signs and symptoms of Parkinson’s disease and may potentially slow its progression.

## 1. Introduction

Parkinson’s disease is the second most prevalent neurodegenerative disease after Alzheimer’s disease, affecting up to 10 million people worldwide and almost 1 million in the USA alone as of 2017 (https://www.parkinson.org/understanding-parkinsons/statistics; URL accessed on 16 November 2023). Parkinson’s disease is the most rapidly increasing neurodegenerative disease worldwide [1] due a longer disease duration and an increasing incidence with age [2]. It is also possible that the current COVID-19 pandemic could further accelerate the number of Parkinson’s disease cases [3,4], along with a worsening of the motor and non-motor signs and symptoms [5]. Parkinson’s disease has a huge social and economic cost, with people with Parkinson’s disease (PwP) living in a state of dependence for many years, at an estimated cost of USD 51.9 billion in 2017, including USD 26.5 billion in care giver, non-medical costs and productivity losses [6]. This is projected to rise to USD 79 billion by 2037.

This review will discuss the current state of Parkinson’s disease diagnosis, pathology, symptoms, treatment and the microbiome–gut–brain axis (MGBA) and will present an argument for the use of photobiomodulation as an adjunct treatment for the symptoms of Parkinson’s disease.

## 2. Diagnosis

Parkinson’s disease is characterised by a range of motor symptoms, although non-motor symptoms are common and may be more disabling. Parkinson’s disease is currently most often diagnosed clinically by symptoms, using patient history, motor signs that are typical of Parkinson’s disease (which are often asymmetric), the lack of atypical features, the exclusion of other diseases, such as atypical parkinsonism (including multiple system atrophy, progressive supranuclear palsy and corticobasal degeneration), vascular parkinsonism, drug-induced parkinsonism and essential tremor. A levodopa challenge test can be used to support a diagnosis of Parkinson’s disease [7]. The accurate diagnosis of idiopathic Parkinson’s disease is made more difficult by the individual nature of the clinical signs and symptoms developed by PwP and the similarity of these symptoms to those displayed by other diseases.

An objective and accurate diagnosis of Parkinson’s disease, especially in the early stages, remains an open question. Less subjective techniques have recently been assessed and in some cases used in clinical practice. These include brain imaging techniques such as functional magnetic brain imaging (fMRI) [8,9], fluorodopa positron emission tomography (F-DOPA PET) and dopamine transporter single photon emission computed tomography (DAT-SPECT) [10,11], as well as the use of artificial intelligence which can compare individual symptoms to datasets of speech, gait, writing and brain scans [12]. A blood, cerebrospinal fluid (CSF) or tissue biomarker would offer the potential of an earlier and more definitive diagnosis of Parkinson’s disease. One such candidate is the presence of aggregated α-synuclein in the enteric nervous system, salivary glands, skin, CSF and extracellular vesicles [13,14]. The α-synuclein seed amplification assay shows great promise in identifying Parkinson’s disease, including subtypes of the disease and early diagnosis in the prodromal period [15]. Other potential biomarkers for the prodromal period of Parkinson’s disease include inflammatory cytokines (such as tumour necrosis factor alpha (TNF-α), interleukin (IL)-1β, and IL-6,) and the non-motor symptoms of REM sleep behaviour disorder (RBD), anosmia and hyposmia, gastrointestinal disturbances and gut microbiome diversity [14]. Diagnosis of RBD may be able to predict progression to Parkinson’s disease decades before clinical diagnosis [16], and almost 10% of people with olfactory dysfunction go on to develop Parkinson’s disease [17,18].

Effective treatment of Parkinson’s disease, if and when available, would be best started as early as possible. Ideally, this would be before current clinical diagnosis, which depends on the establishment of diagnostic protocols that can detect Parkinson’s disease objectively and early.

## 3. Neurodegeneration

Parkinson’s disease is characterised by the progressive loss of dopaminergic neurons, initially in the substantia nigra pars compacta (SNc) in the midbrain and, over a period of many years, progressing to other brain centres [19]. This cell death and loss of function might be related to mitochondrial dysfunction, which has a major influence on Parkinson’s disease, especially in the high energy demand dopaminergic neurons [20]. The initiation and progression of Parkinson’s disease is related to an accumulation of mutations in the mitochondrial DNA, leading to increased ROS production, oxidative stress, mitochondrial damage, disruption of mitochondrial fission and autophagy, and death of the cell [21]. There is some evidence that this mitochondrial damage can spread in the brain via mitochondrial DNA in a prion-like manner [22]. Many of the genes that have been linked to genetically acquired Parkinson’s disease have an effect on the mitochondria [21,23], and the toxins that are risk factors for Parkinson’s disease (see below) also inhibit mitochondrial function [20]. There is also loss of neurons in the olfactory bulb, dorsal motor nucleus of the vagus nerve, pedunculopontine nucleus, the raphe nuclei, the locus coeruleus and also the hypothalamus [24].

The neuronal loss results in ever-lowering levels of the neurotransmitter dopamine in the striatum, resulting in abnormal neuron firing and hence the motor signs of Parkinson’s disease, which become more severe with increased loss of dopaminergic neurons. Eventually, the loss of neurons spread to other areas of the brain, such as the temporal limbic cortex, neocortex and the prefrontal cortex [24]. This process occurs slowly over many years, accounting for the slow progression of Parkinson’s disease symptoms. This loss will also have been occurring many years prior to neurological symptoms becoming apparent. Thus, when neurological symptoms appear, there may have been a loss of between 50% and 70% of dopaminergic neurons in the SNc [25], which suggests that the disease is already at an advanced stage when diagnosed. Thus, a diagnostic tool to detect Parkinson’s disease at earlier stages is critical to manage the disease. Other symptoms of Parkinson’s disease, such as musculoskeletal problems, gastrointestinal issues (see below) and other non-motor symptoms, are often recognised in retrospect.

Parkinson’s disease is also characterised by an accumulation of aggregated α-synuclein within the neurons, called Lewy bodies. Lewy bodies are also present in Lewy body dementia. Alpha-synuclein in its unaggregated form is an intrinsically disordered soluble protein which binds to synaptic vesicles and may control neurotransmitter release [26]. It also may have a role in mitochondrial function [26]. As an aggregated protein, it is thought to spread in a prion-like manner, with aggregated protein seeding further aggregation. This aggregation of α-synuclein has a role in the disease process, as confirmed by animal experiments, and a number of animal models of Parkinson’s disease are based on overexpression of α-synuclein [27]. Increasing α-synuclein aggregation contributes to non-motor symptoms [28].

Inflammation and specifically neuroinflammation also play a role in Parkinson’s disease, although the exact nature of this is unclear. Inflammation may be a consequence of neuronal death, combined with α-synuclein aggregation. Microglia are activated (microgliosis) by both cell death and α-synuclein aggregation in Parkinson’s disease. They migrate to SNc to engulf cellular debris and α-synuclein where they also trigger inflammasome formation and mediate neuroinflammation through the release of pro-inflammatory markers [29], which is a hallmark of all neurodegenerative diseases. Neuroinflammation appears to be linked to disease initiation and progression [30]. Neuroinflammation in Parkinson’s disease is also accompanied by vascular changes in the brain and blood–brain barrier (BBB) leakage, influenced by pro-inflammatory cytokines [31].

Peripheral systemic inflammation is also associated with Parkinson’s disease and has been hypothesised to act as a trigger or risk factor for Parkinson’s disease initiation and/or progression [31]. PwP have increased levels of pro-inflammatory cytokines (TNF-α, IL1-β and IL-6) in serum and CSF, and there are links between peripheral inflammation and neurodegeneration via the BBB and the vagus nerve. Peripheral systemic inflammation is profoundly influenced by the gut microbiota and intestinal permeability (see below). It is also worth noting that dopamine can downregulate peripheral inflammatory processes [32].

Potential treatments for Parkinson’s disease might logically target its pathological mechanisms. Thus, treatments might target mitochondrial function, α-synuclein build up, microglial activation and inflammatory processes. For example, it has been reported that non-steroidal anti-inflammatory drugs (NSAIDs) might somewhat reduce the risk of developing Parkinson’s disease [33].

## 4. The Microbiome–Gut–Brain Axis Connection

It is well established that there is strong MGBA, with a dysfunctional microbiome (dysbiosis) being associated with many neurodegenerative, neurodevelopmental and neuropsychiatric diseases, such as Alzheimer’s disease, multiple sclerosis, amyotrophic lateral sclerosis, multiple system atrophy, autism spectrum disorder, schizophrenia, depression and anxiety, as well as mood and behaviour. This connection is particularly strong in Parkinson’s disease [34].

A healthy microbiome is characterised by high microbial diversity and the abundance of short chain fatty acid (SCFA)-producing bacteria and bacteria producing anti-inflammatory conditions, such as the genera *Faecalibacterium*, *Roseburia*, *Prevotella* and *Dorea*, some *Bacteroides* species, *Clostridium* cluster IV (leptum), and some genera in the order *Lachnospirales.* Upsetting the balance of bacteria in the gut (dysbiosis) can be caused by multiple factors including poor diet, lack of prebiotic fibres, obesity and antibiotics [34]. Typically in dysbiosis, microbial diversity is reduced, healthy bacteria decrease as a proportion of the population or disappear entirely, SCFA levels decrease and there is a concomitant increase in pro-inflammatory bacteria, including those that release lipopolysaccharide (LPS), as well as potentially pathogenic bacteria such as *Enterococcus*, *Megasphaera*, *Desulfovibrio*, *Streptococcus*, *Staphylococcus* and the family Enterobacteriaceae (*E. coli/Shigella*, *Salmonella* and *Klebsiella*) [34].

In Parkinson’s disease, it has been shown in cross-sectional studies that dysbiosis is a characteristic of PwP compared to healthy controls [35,36,37,38,39,40,41,42]. Interestingly, in addition to the typical dysbiosis as seen with other metabolic and neurodegenerative diseases, some bacteria that are often recognised as belonging to a healthy microbiome can be increased in PwP, including *Akkermansia*, *Bifidobacterium* and *Lactobacillus* [43]. Gastrointestinal complaints are very common in PwP, with up to 70% suffering symptoms [44] which include gastric motility issues, constipation, diarrhoea, constipated diarrhoea, faecal incontinence, cramping, bloating and abdominal pain. These symptoms can predate diagnosis by many years [45,46] and may increase with disease [47]. Dysbiosis leads to increased intestinal permeability, increased microbial toxin translocation (especially LPS) and abdominal inflammation, which are common in Parkinson’s disease [48]. Increased inflammation also favours the aggregation of α-synuclein in the enteric nervous system [39], which, at least in animal models, can travel to the brain via the vagus nerve [49,50,51,52]. In humans, α-synuclein aggregation can be detected by biopsy in the gut [53], as well as in the vagus nerve and enteric nervous system [52,54], prior to symptoms of Parkinson’s disease becoming apparent, and truncal vagotomy appears to decrease the risk of Parkinson’s disease [55].

There is good evidence that the association of dysbiosis with Parkinson’s disease might be causative rather than a consequence of the disease and that at least some forms of Parkinson’s disease begin in the gut. This is the “body-first” form of Parkinson’s disease, with α-synuclein aggregation beginning in the gut and traveling to the brain via the vagus nerve [56]. The other form of Parkinson’s disease is hypothesised to be “brain first”, with α-synuclein aggregation beginning in the brain, and REM sleep behaviour disorder occurring in the prodromal period [57]. Epidemiological studies show that a history of constipation [46], IBD [58,59] and IBS [59,60,61] increases the risk of developing Parkinson’s disease, as does the use of some antibiotics [62,63]. Animal studies also show that transplantation of the faeces from PwP worsens Parkinson’s disease signs in mice that overexpress α-synuclein, while faecal transplant from healthy donors does not [64]. The route of environmental toxins through the gut and the proximity of the enteric nervous system might suggest that this exposure over years is a plausible trigger to initiate the cascade that culminates in Parkinson’s disease.

Early treatment of gut problems such as chronic constipation or diarrhoea, IBS and IBD might conceivably alter the progression to Parkinson’s disease later in life [59]. This intervention might be through medication, diet or photobiomodulation.

## 5. Symptoms

Parkinson’s disease is remarkably heterogenous in terms of symptom presentation, with the involvement of multiple body systems. It is characterised by the cardinal motor signs of Parkinson’s disease, which are bradykinesia (slow movement), rigidity, resting tremor and postural instability, but other motor symptoms include gait difficulties (including shuffling gait), freezing, dystonia (involuntary movements), akinesia (lack of movement), facial masking (lack of facial expression), pill-rolling tremor, quiet speech, dysphasia (language impairment), drooling and micrographia (reduced handwriting size). Non-motor symptoms include sleep disturbance, fatigue, pain, gastrointestinal symptoms (diarrhoea and/or constipation, vomiting), excessive drooling, anxiety, depression, apathy, mood disorders, neuropsychiatric disorder (such as hallucinations and vivid dreams), autonomic dysfunction, hyposmia and anosmia (reduction in or loss of sense of smell), urinary urgency/frequency/nocturia/incontinence, erectile dysfunction and (later) cognitive decline. Symptoms of Parkinson’s disease detected early can be very mild, such as slight abnormalities in gait, some clumsiness, slight tremor with the hand at rest, sleep disturbance, hyposmia and centralised pain.

Parkinson’s disease begins before clinical diagnosis. In its pre-clinical stage, pathogenic changes would be occurring that do not present as symptoms. Observable symptoms occur in the prodromal phase of the disease, but these are not necessarily recognised as connected to Parkinson’s disease. These symptoms include RBD, olfactory loss, autonomic dysfunction and gastrointestinal disorders. These can precede clinically diagnosed Parkinson’s disease with the manifest motor and non-motor symptoms, including α-synuclein accumulation, neurodegeneration and neuroinflammation, by up to 20 years. The symptoms associated with the prodromal phase might allow for a prediction of Parkinson’s disease before onset and may also forecast the trajectory of the disease [65]. Early diagnosis of the prodromal phase and even the pre-clinical phase would aid in early treatment of Parkinson’s disease and possibly (with disease-modifying treatment) avoid fully developed Parkinson’s disease.

## 6. Potential Causes

Parkinson’s disease is chiefly known as an idiopathic disease, with its cause still largely unknown. The most important risk factor for Parkinson’s disease is age, with incidence rising sharply above age 65, with men being more susceptible than women (in a ratio of approximately 3:2). There is a genetic component to the disease, which accounts for between 10 and 15% of cases, with several individual genes (such as LRRK2, SNCA, PINK1, PARK7 and PRKN) associated with Parkinson’s disease [66] and over 90 gene loci that can increase the risk of developing Parkinson’s disease [67].

There is increasing evidence that other cases of idiopathic Parkinson’s disease may be related to environmental toxins [68,69,70], including pesticides (such as rotenone and permethrin [71,72]), herbicides (such as paraquat and 2,4-dichlorophenoxyacetic acid [72,73]), ubiquitous organic pollutants (such as polychlorinated biphenyls [74]), solvents (such as trichloroethylene [1]) and possibly heavy metals (mercury [75]). The herbicide glyphosate has received much attention in recent years as a potential risk factor for Parkinson’s disease [76,77]. This is especially relevant to Parkinson’s disease risk due to its ubiquitous use as a herbicide in agriculture, its presence in soil, water, plants, animals, and foods, its detection in human urine, blood and breast milk [77] and the application for a patent to use the chemical as an antibiotic (https://patents.google.com/patent/US7771736B2/en). Despite efforts to ban its use, most countries continue to use the herbicide and the European Union voted to renew its approval on 16 November 2023 (https://ec.europa.eu/commission/presscorner/detail/en/qanda_23_5793). Many animal models of Parkinson’s disease are based on toxins, including the synthetic (non-toxic) compound 1-methyl-4-phenyl-1,2,3,6-tetrahydropyridine (MPTP), which is converted to the toxic metabolite 1-methyl-4-phenylpyridinium (MPP^+^), and the pesticide rotenone, both of which poison the electron transport chain of the mitochondria.

In addition, there are life style and behavioural factors which can increase Parkinson’s disease risk, such as head trauma [78], mental illness [75] and the consumption of dairy products [79], or decrease Parkinson’s disease risk, such as exercise [80], tobacco [81] and coffee [82]. There are also a number of diseases and conditions that are known to increase the risk of developing Parkinson’s disease. These include type II diabetes [83], gastrointestinal conditions such as irritable bowel syndrome (IBS) [61], inflammatory bowel disease IBD [59] and antibiotic use [62,63], as well as conditions that are known to be associated with the prodrome of Parkinson’s disease [84], such as constipation, loss of sense of smell (4–6 years prior to motor signs), idiopathic insomnia and RBD, with a 45% risk of progressing to Parkinson’s disease in 5 years and 76% in 10 years [14].

Identifying risk factors and potential causes of Parkinson’s disease might assist in developing treatments for the disease. For example, it is possible that glucose-lowering drugs used to control symptoms of type II diabetes might reduce the risk of developing Parkinson’s disease [85].

## 7. Pharmacological Treatment

Treatment for Parkinson’s disease is currently a matter of treating symptoms. There has been no cure or preventative therapy for the disease and there is no medication to treat the pathology, provide neuroprotection or modify the trajectory of the disease. The heterogeneity of the disease creates a challenge for treatment, resulting in a cocktail of medications to chase individual signs and symptoms. Treatment focuses on improving motor symptoms such as tremors, rigidity and bradykinesia, using dopamine-based pharmacological treatments. Most PwP are initially treated with dopamine replacement therapy to substitute for the dopamine loss caused by neuron death. L-dopa given orally can pass the BBB (unlike dopamine) and is converted to dopamine in the brain. L-dopa is often given together with carbidopa to limit peripheral transformation of L-dopa to dopamine. L-dopa can be effective in reducing motor signs, but not all PwP respond to the medication and not all clinical signs respond equally. Tremors and balance tend to be least responsive, and rigidity and bradykinesia respond the most. While initially effective, with prolonged use, the efficacy L-dopa is reduced, resulting in the need to increase dosages. L-dopa also has pronounced “on” periods where the dopamine replacement is working well to relieve symptoms and “off” periods between medications. With prolonged use, the “on” periods become shorter. L-dopa can also have a number of side effects, some of which can be serious. These include worsening symptoms, dyskinesia, cardiac arrythmia, nausea, orthostatic hypotension, dizziness, daytime somnolence, confusion, hallucinations, delusions, psychosis, agitation, addiction (e.g., gambling), hypersexuality, hypomania, nocturnal hyperactivity and punding (a fascination with repetitive examination and handling of objects, e.g., compulsive sorting and arranging objects, taking apart mechanical objects, etc.) [86]. These side effects affect PwP to different extents. Dyskinesia might be reduced by the addition of amantadine, anticholinergics or beta-blockers.

The changing “on-off” periods, the extent of side effects and the waning of effectiveness over time requires continual individual titration of L-dopa dose. In more advanced Parkinson’s disease, where motor fluctuations cannot be controlled with optimising oral L-dopa, percutaneous endoscopic gastrostomy with a jejunal extension (PEG-J) may be considered for continuous delivery of L-dopa–carbidopa intestinal gel.

Other dopamine replacement pharmaceuticals include dopamine agonists, which mimic dopamine, and monoamine oxidase B inhibitors. Dopamine agonists can have fewer and less serious side effects than L-dopa, but these can still be significant and include drowsiness, insomnia, nausea, vomiting, orthostatic hypotension, headache, dizziness, cardiac arrhythmia, hallucinations, delusions, confusion, depression, constipation, neuropsychiatric episodes and impulse control disorders [87].

Monoamine oxidase B (MOAB) inhibitors and catechol-O-methyl transferase (COMT) inhibitors block the enzymes that inactivate or convert dopamine in order to extend its half-life in the body. These may help the on–off periods, especially in the early stages of the disease. Side effects of MOAB inhibitors can include mild nausea, dry mouth, light-headedness, constipation and confusion and hallucinations in elderly PwP. Side effects of COMT inhibitors can include increased side effects of L-dopa, confusion, hallucinations, diarrhoea and discoloured urine. Amantadine can help control tremors and L-dopa-induced dyskinesia. Side effects can include dizziness, low blood pressure, nausea, insomnia, confusion, hallucinations, paranoia, urinary retention and leg discolouration.

When L-dopa has lost its effectiveness, PwP can be recommended for deep brain stimulation (DBS), which implants electrodes and targets the subthalamic nucleus with electrical stimulation to compensate for the lack of dopamine and re-organise the brain’s electrical signals in the centre that controls movement. Tremors, stiffness and slow movement are the most successfully controlled clinical signs. Side effects of DBS include the normal risks of invasive surgery and implanted devices (infection, bleeding in the brain, heart and lung problems, stroke, seizures and pain), numbness, muscle tightness, headaches, speech difficulties, balance problems, vision changes, hypersexuality, hallucinations, mood changes (apathy, anger, uncontrolled crying, depression, suicide and suicidal ideation) and personality changes [88].

The list of pharmaceutical interventions for non-motor symptoms is extensive [89] and includes selective serotonin inhibitors (SSIs) and benzodiazepines for depression, cholinesterase inhibitors for cognition and laxatives for constipation, each with its own list of side effects.

There is currently no treatment that can cure Parkinson’s disease or halt or even slow the inevitable progression of clinical signs or symptoms of the disease. There is a need for new treatment modalities as an adjunct to pharmacological interventions.

## 8. Photobiomodulation as a Potential Treatment for Parkinson’s Disease

Photobiomodulation is the use of light energy to modulate cellular responses. The light source can be a laser or LEDs of specific wavelengths, with an energy below 500 mW producing a molecular effect that is non-thermal. PBM is absorbed by chromophores that change their conformation and thus produce a biochemical and a cellular effect. The most important chromophore is cytochrome-C-oxidase (CCO), complex IV in the electron transport chain in the mitochondria. Absorption of specific wavelengths of light (red and near infrared) cause the CCO to increase the flow of protons across the inner membrane (increasing the mitochondrial membrane potential), release reactive oxygen species (ROS) and increase the binding of oxygen, all of which, in turn, increase ATP production, as well as modulating downstream cellular signalling and gene transcription via cAMP, NO and ROS [90,91,92,93]. PBM also interacts with transient receptor potential vanillin (TRPV) channels, which undergo conformational change and facilitate a range of cellular effects [94]. PBM has proven to be a safe and non-invasive treatment for many conditions. PBM has been used for over five decades in clinical practice to heal tissues, including wounds, burns and chronic ulcers, to relieve pain, to repair deep tissues such as tendons, cartilage and bones, and to reduce inflammation in conditions such as osteoarthritis, endothelial dysfunction and chronic pain [95,96,97]. Over this period, PBM has a remarkable safety record with vanishingly small numbers of safety or adverse effects for any condition including cancers [98].

Most recently, transcranial PBM has been used with increasing frequency to target the brain for trauma (traumatic brain injury (TBI) and stroke), neurodegenerative diseases and neuropsychological conditions. The penetration depth of near-infrared wavelengths of PBM has been shown to be of the order of centimetres [99], which is sufficient to reach the brain. The external (transcranial) delivery of PBM in animal models has been shown to be effective in pre-clinical models of TBI, stroke, Alzheimer’s disease, ALS and MS in reducing symptoms, to be neuroprotective and to regenerate neurons [100,101,102,103,104]. In humans, transcranial PBM has been trialled, with varying success, for cognitive improvement [105,106] and to treat ischemic stroke [107,108], traumatic brain injury [109,110,111], CTE [112], PTSD [113], anxiety and depression [114,115], Alzheimer’s disease [116,117], autism spectrum disorder [118,119] and addiction to opioids [120]. The use of transcranial PBM (laser and LED) has also proven to be extraordinarily safe with very few side effects, which are invariably minor and transient [121,122,123].

### 8.1. In Vitro Experiments

Parkinson’s disease, as with a number of other neurodegenerative diseases, has characteristics of inflammation (neuroinflammation), oxidative stress and mitochondrial dysfunction. Since these are cellular characteristics that PBM is known to modulate, it follows that PBM might have a positive effect on this disease, given the correct anatomical target and dose. There is, in fact, compelling pre-clinical experimental evidence that PBM can modify Parkinson’s disease.

In vitro experiments using a 670 nm LED light source to irradiate striatal and cortical neurons poisoned with Parkinson’s disease-inducing toxins MPP^+^ and rotenone (as well as KCN) demonstrated reversal of the effects of the toxin with increased CCO activity, increased ATP production, reduced ROS production and the inhibition of apoptosis [124]. The effect was also apparent in neurons that were pre-treated with LED before administration of the toxin [125]. The same group also showed that human dopaminergic neuronal cells that overexpress α-synuclein, exposed to MPP^+^, had a dose-dependent decrease in viability and proliferation, mitochondrial function and oxidative stress [126]. Since this time, PBM at 810 nm has been shown to restore mitochondrial transport in a cytoplasmic hybrid model of PBM (incorporating mitochondrial DNA from Parkinson’s disease patients) [127]; PBM at 633 nm and 840 nm has been shown to stimulate oxygen consumption and increase cell viability in a rotenone in vitro model of Parkinson’s disease [128]; both a 635 nm laser and curcumin treatment have resulted in reduced oxidative stress and reduced cell death compared to untreated cells in a 6-OHDA toxin, PC12 cell model of Parkinson’s disease [129]; a 632.8 nm laser has been found to be neuroprotective and reverse the loss of vesicular monoamine transporter 2 expression in a human neuroblastoma cell model using MPP^+^ as the toxin, hence promoting dopamine transport [130]. The effect of PBM in these studies was to protect neurons from Parkinson’s disease-related toxins and insults.

### 8.2. In Vivo Transcranial Experiments

The first in vivo experiments using PBM for Parkinson’s disease began with a collaboration between the Mitrofanis and Stone Labs at Sydney University, showing in 2010 that treatment with 670 nm LEDs can reduce Parkinson’s-like symptoms and reduce dopaminergic neuronal cell death (neuroprotection) in an MPTP mouse model [131]. This effect has been since demonstrated many times in this lab using the MPTP mouse model [132,133,134,135,136,137,138,139,140], as well as a transgenic mouse model that overexpressed hyperphosphorylated tau 670 nm [141], a 6-OHDA rat model [142] and an MPTP monkey model [143,144,145,146,147] (Table 1). The positive effect of treatment with PBM has also been demonstrated in other labs with MPTP-treated mice [126], 6-OHDA-treated rats [148,149], LPS-treated rats [150] and rats that overexpress α-synuclein [27]. Common to all studies is the neuroprotective effect of PBM, with significantly more surviving cells than untreated animals. PBM applied to the brain has also been shown to reduce gliosis [145], reduce oxidative stress [141], reduce TNF-α [149], reduce toxicity of α-synuclein [27], reduce vascular leakage [133], reduce Fos+ cell numbers (that are increased with Parkinson’s disease models) [140] and improve the mobility and behaviour of the animals [126,134,136,137,141,147,149,150]. In a Drosophila model of Parkinson’s disease, using a PINK 1 mutation that produces mitochondrial dysfunction, it was shown that illumination with a 808 nm laser stimulated CCO to improve mitochondrial function, but there was no increase in ATP due to the rate-limiting step being the mutation that affects Complex I [151].

Both red (630 nm; 670 nm [126,131,133,134,135,136,137,138,139,140,141,144,145,147,149,150]) and infrared (808 nm; 810 nm [27,136,151]) wavelengths can be neuroprotective, with both LED [126,133,134,135,136,137,138,139,141,144,145,149] and laser [27,149] light sources. The effect is dose-dependent, with more energy required for higher MPTP doses [135] but generally lower irradiances having a greater effect than higher irradiances [27]. The neuroprotective effect occurred whether the PBM was applied before, at the same time or after the toxin insult [132,134,142] and could last for some weeks after the cessation of PBM treatment in rodents [27] and monkeys [147].

### 8.3. In Vivo Remote Experiments

The remote or systemic effect of PBM is a known consequence of PBM treatment to one part of the body, causing a (distal) effect in another part of the body [91,152,153,154,155]. The mechanism of this effect is usually assumed to be cytokines or other chemical messengers [156] or stem cell activation [154]. In the Parkinson’s disease animal model, the Stone/Mitrofanis/Johnstone lab showed that irradiation of mice with 670 nm LED, when the head was shielded, produced similar (although lesser) neuroprotection compared to transcranial irradiation [152,157] (Table 1), suggesting a similar mechanism for both forms of irradiation. Pre-conditioning with remote PBM also produced a neuroprotective response to MPTP, similar to that seen with remote ischemic pre-conditioning, and interestingly, a combination of the two pre-conditioning regimens did not increase the neuroprotection [158]. Pre-conditioning PBM to the dorsum and hind limbs of mice was found to reduce oxidative stress and possibly protect the BBB from subsequent damage by MPTP [132]. Gordon et al. [143] showed that PBM (656 nm LED), directed to the abdomen or legs of mice on the day following MPTP treatment, resulted in neuroprotection similar to PBM directed to the head. Interestingly, in a non-human primate study conducted on single animals, remote PBM to the abdomen or legs showed a pronounced reduction in the clinical signs of Parkinson’s disease compared to MPTP controls that exceeded transcranial PBM. Potential mechanisms of this remote PBM effect include activation of stem cells (either in the bone marrow of the tibia or in the adipose tissue of the abdomen), circulating cytokines, circulating free-floating active mitochondria, circulating mitokines or other enteric messengers, and stimulation of the gut–brain axis via the vagus nerve, or the gut microbiome [143,159], which has been reported to be modified by PBM in the abdomen of mice [160].

Laser acupuncture is also a form of remote application of light, although the mechanism of action is likely to be entirely different. Laser acupuncture with 405 nm to point HT7 (at the wrist) in a 6-OHDA rat model was seen to reduce neural degeneration and memory loss [148].

The use of remote treatment for Parkinson’s disease appears to be a viable alternative to direct transcranial PBM in animal models [161].

### 8.4. Clinical Trials

There are few clinical studies that have addressed PBM as a therapy for Parkinson’s disease symptoms (Table 2). The translation of animal studies to clinical trials is fraught with difficulties, with the result that there are vanishing small numbers of successful therapies for neuroprotection generally [162,163]. This includes for Parkinson’s disease [164], where no neuroprotective medication has as yet been identified. For example, despite the success in pre-clinical models for chelators to scavenge excess iron and for coenzyme Q10 to repair mitochondrial defects and oxidative stress, there is no evidence of their clinical usefulness [165].

It has been reported that some studies using light therapy for Parkinson’s disease have been conducted prior to 2005 in Chinese and Russian languages [166]. These studies have used He-Ne (632.8 nm) intranasal or intravenous lasers and have apparently produced promising results. In 2010, an abstract of the American Society for Laser Medicine and Science [167] reported results for a non-randomised Parkinson’s disease study that used two weeks of transcranial PBM and assessed a range of motor and non-motor outcomes using a visual analogue scale (VAS). While few details were given, significant improvement was evidently seen in gait, speech, freezing and cognition [168].

Santos et al. [169], in a letter to the editor, reported positive results for gait speed (ten-metre walk test (10MWT) at fastest possible speed) in a randomised placebo-controlled clinical trial (RCT) with 35 participants, using transcranial 670 nm LED (twice per week for 9 weeks) aimed at the left and right temple region. No other outcome measures (MDS-UPDRS, posture, timed up-and-go (TUG) and 10MWT at preferred speed) were reported as significantly altered.

Hamilton et al. [170,171] have described, as case studies, six patients with Parkinson’s disease that have been treated with a variety of “buckets” or helmet devices lined with LEDs of various wavelengths (670 nm, 810 nm, 850 nm and 940 nm) or an intranasal device (660 nm) for up to 24 months. These participants have shown a variety of motor and non-motor improvements (both subjective and objective) including micrographia, reduced tremors, akinesia, improved gait, reduced facial masking, improved sleep, improved speech, increased motivation and improvements in their sense of smell.

Berman and Nichols [172] reported some results for the treatment of Parkinson’s disease patients with dementia (1068 nm LED, twice daily), with two participants showing improvements in clock drawing.

A small trial (18 participants) compared PBM therapy (laser, 670 nm + 808 nm) with vacuum therapy and vacuum plus PBM therapy (twice per week for 3 weeks) [173]. The outcome measures were pain, assessed using VAS and a quality-of-life questionnaire. The results suggested that the combination of PBM and vacuum therapy provided more pain relief and improvement in quality of life than the two therapies alone. There was, however, no control group.

In a proof-of-concept study, Liebert et al. [174] conducted a waitlist design clinical trial, with six participants beginning immediately and six participants waiting for 14 weeks before treatment began. Treatment consisted of a transcranial LED (810 nm), an intranasal LED (810 nm) and an abdominal and neck laser (904 nm), three times per week for 12 weeks, followed by at-home treatment for one year. The abdominal treatment was designed to take advantage of the remote treatment effect described in animal studies [156]. Movement outcome measures included TUG, TUG motor, TUG cognitive, 10MWT, dynamic balance (step test), static balance (single-leg stance and tandem stance), spiral test, micrographia (writing test) and nine-hole peg test. Non-motor outcome measures included Montreal Cognitive Assessment (MoCA). While numbers were small and the study lacked a control group, there were significant improvements at 12 weeks in TUGs, walk speed and stride length, dynamic balance, spiral test and MoCA. Apart from walk speed and stride length, these improvements were maintained for one year. There was also a discernible Hawthorne effect as a result of being part of a clinical trial. Participants showed a great variety in their symptoms and their improvements, with the majority showing clinically significant improvements in multiple outcome measures. When re-tested after 3 years, those participants who continued treatment had, on the whole, maintained their motor and MoCA improvements [175], with some participants continuing to improve for some outcome measures such as static balance. There was also an indication that if treatment was discontinued, many of the improvements in symptoms were lost [175], suggesting that as a chronic neurodegenerative disease, treatment needed to be maintained, perhaps in perpetuity.

Bullock-Saxton et al. [176] conducted an RCT with 22 participants that used a laser (904 nm) on four points transcranially and one point at the junction of the soft and hard palates, one, two or three times per week for 4 weeks. They found changes (although non-significant) in a positive direction in several outcome measures, including the spiral test and dynamic balance.

In a study using PBM and hydrogen water [177], 18 participants received daily hydrogen water (200 mL) and PBM (940 nm LED, daily each weekday for 2 weeks) placed at the neck and directed towards the mid-brain. The outcome measure was UPDRS (combined parts I, II and III), which showed a significant change in total UPDRS score after one and two weeks of treatment, due mainly to part I of the UPDRS (non-motor aspects of experiences of daily living).

A study [178] comparing standard care physiotherapy (n = 26) with physiotherapy plus PBM therapy (n = 12) using a transcranial helmet (256 × 810 nm LEDs, for 18 min, twice per week for four weeks) found that all parts of the MDS-UPDRS improved for both groups, although the physiotherapy plus PBM group showed a greater response that also persisted for one month without treatment. Unfortunately, there was no control group in this study.

A recent RCT with 20 + 20 participants [179,180,181] compared a placebo helmet with an active transcranial helmet (20 × 635 nm LEDs + 20 × 810 nm LEDs, 12 min of red followed by 12 min infrared per day, 6 days per week, for 12 weeks). The trial was conducted in the participant’s own home, with treatment performed by the participant and/or care giver. Outcome measures including a modified MDS-UPDRS part III (motor) were assessed using internet conferencing. At the end of the 12 weeks, the participants were unblinded and the placebo group was given the opportunity to switch to the active helmet for a further 12 weeks and the active group received no treatment. The PBM treatment proved to be both safe and feasible to deliver as an adjunct therapy [180]. Both groups showed significant improvement in the modified MDS-UPDRS motor score after 12 weeks. When the placebo group switched to the active helmet, there was continued improvement in motor scores for up to 24 weeks. In addition, when sub-scores within the motor part of the MDS-UPDRS for the first 12 weeks were examined, there appeared to be a greater improvement for responders to the PBM treatment compared to responders to the placebo effect [181].

### 8.5. PBM Penetration to the Substantia Nigra and Remote PBM Treatment

While the animal models and the limited clinical studies are promising, a major hurdle for the use of PBM to the target symptoms of Parkinson’s disease is the delivery of light to the appropriate area of the brain, as clearly articulated by Mitrofanis [182]. Neuronal death begins in the SNc. While transcranial penetration of PBM to the SNc in rodents is not a problem, it becomes a major concern in humans and larger animals. Transcranial PBM needs to penetrate the human hair, skin, tissue, bone, dura, blood, meninges and CSF to reach the neurons in the brain [99]. The maximum depth of penetration transcranially is most probably in the order of 20 to 30 mm, with progressively fewer photons with increasing depth [123]. PBM light reaching the SNc in humans where most damage is occurring is not physically possible. In an effort to overcome this problem, some studies have attempted to target the PBM towards the SNc by placing the laser on the palate [176], using intranasal devices [171] or by placing the LED on the mid-line of the neck, pointed toward the midbrain [177].

To address the penetration problem, one group has investigated the use of implanted light devices to deliver light to the correct brain centres (Table 1). Light delivery fibre optics (both LED and laser) have been implanted in mice [183], rats [142] and monkeys [144,146,147,184,185], with no adverse effects and with improvements in clinical signs and neuroprotection. Again, there was a dose effect, with increased energy doses showing reduced neuroprotection [146].

**Table 1:** Summary of studies investigating the effect of photobiomodulation in animal models of Parkinson’s disease.

Study	Animal Model	Design	PBM Parameters	Outcomes
**TRANSCRANIAL PBM**				
Shaw et al., 2010 [131]	Mouse; BALB/c males; MPTP model.	Saline (n = 20)/saline + PBM (n = 20)/MPTP (n = 20)/MPTP + PBM (n = 20).	670 nm LED; Transcranial; 40 mW/cm^2^ for 90 s; Four treatments over 30 h (after MPTP).	Reduced neuronal loss; Increased TH^+^ cell numbers in SNc.
Shaw et al., 2012 [140]	Mouse; BALB/c males; MPTP model.	Saline (n = 24)/saline + PBM (n = 24)/MPTP (n = 24)/MPTP + PBM (n = 24).	670 nm LED; Transcranial; 40 mW/cm^2^ for 90 s; Four treatments over 30 h (after MPTP).	Reduced c-FOS expression in both acute and chronic models.
Peoples et al., 2012 [138]	Mouse; BALB/c males; MPTP model.	Saline (n = 21)/saline + PBM (n = 19)/MPTP (n = 22)/MPTP + PBM (n = 18).	670 nm LED; Transcranial; 40 mW/cm^2^ for 90 s; Ten treatments after each MPTP injection OR Ten treatments after 3 weeks.	Increased TH^+^ cell numbers in SNc for both simultaneous PBM treatment and post-treatment.
Peoples et al., 2012 [139]	Mouse; BALB/c males; MPTP model.	Saline (n = 21)/saline + PBM (n = 19)/MPTP (n = 22)/MPTP + PBM (n = 18).	670 nm LED; Transcranial; 40 mW/cm^2^ for 90 s; Four treatments 4 days after MPTP (simultaneous treatment model) OR Ten treatments over 3 weeks (post-treatment model).	Increased TH^+^ cell numbers in the retina for both simultaneous PBM treatment and post-treatment.
Moro et al., 2013 [137]	Mouse; BALB/c males + C57BL/6; MPTP model.	Saline (n = 10 + 10)/saline + PBM (n = 10 + 10)/MPTP (n = 10 + 10)/MPTP + PBM (n = 10 + 10).	670 nm LED; Transcranial; 40 mW/cm^2^ for 90 s; Two PBM treatments immediately after and 6 h after each MPTP injection over 30 h (total of four PBM treatments).	ncreased TH^+^ cells in SNc and improved locomotion for albino (BALB) mice but not for black (C57BL) mice—due to reduced penetration of 670 nm.
Purushothuman et al., 2013 [141]	Mouse; K3 transgenic (overexpression of tau protein).	Wild type (n = 5)/K3 (n = 5)/K3 + PBM (n = 5).	670 nm LED; Transcranial; 40 mW/cm^2^ for 90 s; One PBM treatment per day, 5 days per week for 4 weeks (total of 20 PBM treatments).	Increased TH^+^ cells in SNc; decreased oxidative stress markers.
Johnstone et al., 2014 [152]	Mouse; BALB/c males; MPTP model (50, 75 and 100 mg/kg).	50 mg/kg MPTP (n = 36)/75 mg/kg MPTP (n = 8)/100 mg/kg MPTP (n = 9)/50 mg/kg MPTP + transcranial PBM (n = 12)/75 mg/kg MPTP + transcranial PBM (n = 8)/100 mg/kg MPTP + transcranial PBM (n = 19)/50 mg/kg MPTP + remote PBM (n = 11)/75 mg/kg MPTP + remote PBM (n = 8)/100 mg/kg MPTP + remote PBM (n = 9).	670 nm LED; Transcranial OR remote; 40 mW/cm^2^ for 90 s; Two PBM treatments immediately after and 6 h after each MPTP injection (total of four PBM treatments for 50 mg/kg, six for 75 mg/kg and eight for 100 mg/kg MPTP).	Increased TH^+^ cells in 50 mg/kg MPTP treatment (not 75 or 100 mg/kg); Transcranial treatment appeared superior to remote treatment.
Oueslati et al., 2015 [27]	Rat; Transgenic overexpression of α-synuclein.	Sham (n = 9)/low PBM (n = 7)/high PBM (n = 7).	808 nm laser; Transcranial; (Two spots); 2.5 J and 5 J; Daily for 28 days.	Reduced neuronal loss; Reduced motor impairment; Improvements lasted for 6 weeks after treatment was discontinued.
Reinhart et al., 2015 [136]	Mouse; BALB/c males; MPTP model.	Saline (n = 11)/saline + PBM (n = 11)/MPTP (n = 11)/MPTP + PBM (n = 11).	810 nm LED; Transcranial; 1.6 mW for 90 s; Four treatments over 30 h (after MPTP).	Increased TH^+^ cells in SNc; Improved locomotion after each PBM treatment.
El Massri et al., 2016 [135]	Mouse; BALB/c males; MPTP model.	Saline (n = 20)/saline + PBM (n = 30)/MPTP (n = 30)/MPTP + PBM (n = 40).	670 nm LED; Transcranial; 40 mW/cm^2^ for 90 s; Immediately after and 6 h after two MPTP injections (four PBM treatments) OR Immediately after and 6 h after four MPTP injections, separated by 7 days (eight PBM treatments) OR Two PBM treatments per day for 8 or 12 days (sixteen PBM treatments).	Increase in TH^+^ cells in 8 PBM treatment group and 16 PBM treatment group and after 7 days in the 4 PBM treatment group but not after 2 days; 2 J/cm^2^ was not effective, while 4 J/cm^2^ was effective for an increased MPTP dose (100 mg/kg).
Ribeiro et al., 2016 [149]	Rat; Wistar; 6OHDA model.	6OHDA (n = 20)/6OHDA + LED (n = 20)/6OHDA + laser (n = 20).	672 nm LED; Right cervical region over carotid artery; 70 mW for 57 s; daily for 7 days; OR 630 nm laser; Right cervical region over carotid artery; 45 mW for 88 s; Daily for 7 days.	Increased IL-2, IL-10 and interferon with a laser; Increased IFN-α with LEDs; Decreased TNF-α with LEDs and a laser; Motor condition improved significantly with a laser but not LEDs.
Reinhart et al., 2016 [134]	Mouse; BALB/c males; MPTP model.	Saline (n = 9)/saline + PBM (n = 11)/MPTP (n = 9)/MPTP + pre MPTP injection PBM (n = 9)/MPTP + simultaneous MPTP injection PBM (n = 9)/MPTP + post MPTP injection PBM (n = 9)/MPTP + pre + simultaneous MPTP injection PBM (n = 9)/MPTP + post + simultaneous MPTP injection PBM (n = 9)/MPTP + pre + post + simultaneous MPTP injection PBM (n = 9).	670 nm LED; Transcranial; 40 mW/cm^2^ for 90 s; Two PBM treatments per day; Pre, post and simultaneous MPTP injection had four PBM treatments; pre + simultaneous and post + simultaneous MPTP injection had eight PBM treatments; pre + post + simultaneous MPTP injection had twelve PBM treatments.	Increased TH^+^ cells in all groups; Locomotion improved in all groups.
San Miguel 2019 [133]	Mouse; C56BL/6 (head shaved); MPTP model.	Saline (n = 8)/MPTP (n = 6)/MPTP + PBM (n = 6).	675 LED; Transcranial; 50 mW/cm^2^ for 3 min daily for 7 days.	Reduced vascular leakage in SNc and the caudate putamen complex.
O’Brien and Austin 2019 [150]	Rat; Sprague–Dawley male; Supranigral LPS injection.	Vehicle sham/LPS sham/LPS + PBM.	670 nm LED array; Transcranial; 4 J/cm^2^ twice daily for 6 days.	
**INTRACRANIAL PBM**				
Moro et al., 2014 [183]	Mouse; BALB/c males; MPTP model.	Saline (n = 5)/saline + continuous PBM (n = 5)/ saline + pulsed PBM (n = 5)/MPTP + continuous PBM (n = 5)/MPTP + pulsed PBM (n = 5).	670 nm LED; Implanted intracranially; 1.5 mW/cm^2^ (pulsed) for 90 s per day for 4 days; 14.5 mW/cm^2^ (continuous) for 6 days continuously.	Pulsed group showed increased TH^+^ cells in SNc; Continuous group showed non-significant increase in TH^+^ cells in SNc.
Moro et al., 2016 [146]	Monkey (macaque); Adult male; MPTP model.	Control (n = 3)/MPTP (n = 5)/MPTP + PBM (n = 7).	670 nm laser; Implanted intracranially; 10 mW on for 5 s/off for 30 s; Continuous for 25 days.	Increased TH^+^ cells but no increased effect of increased dose compared to Darlot et al. [147].
Reinhart et al., 2016 [142]	Rat; Wistar; 6OHDA model.	Saline (n = 8)/6OHDA (n = 15)/6OHDA + PBM (pulsed) (n = 16)/6OHDA + PBM (continuous) (n = 13)/6OHDA + PBM (continuous low dose) (n = 9).	670 nm LED Implanted intracranially; 1.6 mW for 90 s twice daily for 23 days OR 1.6 mW continuously for 23 days OR 0.333 mW continuously for 23 days.	Increased TH^+^ cells for pulsed group but not continuous groups; Decreased rotational behaviour for pulsed and high-dose continuous groups but not for low-dose continuous group.
Darlot et al., 2016 [147]	Monkey (macaque); Adult male; MPTP model.	Control (n = 5)/1.5 mg/kg MPTP (n = 6)/2.1 mg/kg MPTP (n = 5)/1.5 mg/kg MPTP + PBM (n = 5)/21.1 mg/kg MPTP + PBM (n = 4).	670 nm laser; Implanted intracranially; 10 mW on for 5 s/off for 30 s; Continuous for 5 days (1.5 mg/kg MPTP) OR Continuous for 7 days (2.1 mg/kg MPTP).	Increased TH^+^ cells; Increased striatal TH^+^ terminals for lower MPTP group; Improved clinical scores; Improved locomotion.
El Massri et al., 2016 [145] Further results of study from Darlot et al., 2016 [147]	Monkey (macaque); Adult male; MPTP model.	Control (n = 5)/1.5 mg/kg MPTP (n = 6)/2.1 mg/kg MPTP (n = 5)/1.5 mg/kg MPTP + PBM (n = 5)/21.1 mg/kg MPTP + PBM (n = 4).	670 nm laser; Implanted intracranially; 10 mW on for 5 s/off for 30 s; Continuous for 5 days (1.5 mg/kg MPTP) OR Continuous for 7 days (2.1 mg/kg MPTP).	Reduced MPTP-induced astrogliosis in the SNc and striatum.
El Massri et al., 2017 [144] Further results from studies of Moro et al. [183], Reinhart et al., 2016 [142] and Darlot et al., 2016 [147]	Mouse; BABL/c. + Rat; Wistar. + Monkey (macaque). All MPTP models.	Mouse: Saline (n = 5)/saline + continuous PBM (n = 5)/saline + pulsed PBM (n = 5)/MPTP + continuous PBM (n = 5)/MPTP + pulsed PBM (n = 5). Rat: Saline (n = 8)/6OHDA (n = 15)/6OHDA + PBM (pulsed) (n = 16)/6OHDA + PBM (continuous) (n = 13)/6OHDA + PBM (continuous + low dose) (n = 9). Monkey: control (n = 5)/1.5 mg/kg MPTP (n = 6)/2.1 mg/kg MPTP (n = 5)/1.5 mg/kg MPTP + PBM (n = 5)/21.1 mg/kg MPTP + PBM (n = 4).	670 nm LED; Implanted intracranially; Mouse: 1.5 mW/cm^2^ (pulsed) for 90 s per day for 4 days; 14.5 mW/cm^2^ (continuous) for 6 days continuously. Rat: 1.6 mW for 90 s twice daily for 23 days OR 1.6 mW continuously for 23 days OR 0.333 mW continuously for 23 days. Monkey: 10 mW on for 5 s/off for 30 s; Continuous for 5 days (1.5 mg/kg MPTP) OR continuous for 7 days (2.1 mg/kg MPTP).	Increase in TH^+^ cells in the striatum of monkeys but not mice or rats; Increase in glial cell-derived neurotrophic factor (GDNF) in monkeys.
El Massri et al., 2018 [184]	Monkey (macaque); Adult male; MPTP model.	Control (n = 3)/control + PBM (n = 3)/MPTP (n = 3)/MPTP + PBM (n = 3).	670 nm laser; Implanted intracranially; 10 mW on for 5 s/off for 60 s; Continuous for 5 days.	No effect on encephalopsin expression in striatum.
**REMOTE PBM**				
Stone et al., 2013 [157]	Mouse; BALB/c males; MPTP model.	Saline (n = 5)/saline + PBM (n = 5)/MPTP (n = 5)/MPTP + PBM (n = 5).	670 nm LED; Remote (body with head shieled); 40 mW/cm^2^ for 90 s; Twp PBM treatments immediately after and 6 h after each MPTP injection over 30 h (total of four PBM treatments).	Increased TH^+^ cells in SNc.
Wattanathorn and Sutalangka 2014 [148]	Rat; Wistar; 6OHDA model.	Control (n = 12)/6OHDA (n = 12)/6OHDA sham (n = 12)/6OHDA + PBM (n = 12).	405 nm laser; Laser acupuncture point HT7; 100 mW for 10 min; Once daily for 14 days.	Improved memory; increased cells at CA3 of the hippocampus; decreased markers of oxidative stress.
Johnstone et al., 2014 [152]	Mouse; BALB/c males; MPTP model (50, 75 and 100 mg/kg).	50 mg/kg MPTP (n = 36)/75 mg/kg MPTP (n = 8)/100 mg/kg MPTP (n = 9)/50 mg/kg MPTP + transcranial PBM (n = 12)/75 mg/kg MPTP + transcranial PBM (n = 8)/100 mg/kg MPTP + transcranial PBM (n = 19)/50 mg/kg MPTP + remote PBM (n = 11)/75 mg/kg MPTP + remote PBM (n = 8)/100 mg/kg MPTP + remote PBM (n = 9).	670 nm LED; Transcranial OR remote; 40 mW/cm^2^ for 90 s; Two PBM treatments immediately after and 6 h after each MPTP injection (total of four PBM treatments for 50 mg/kg, six for 75 mg/kg and eight for 100 mg/kg MPTP).	Increased TH^+^ cells in 50 mg/kg MPTP treatment (not 75 or 100 mg/kg); Transcranial treatment appeared superior to remote treatment.
Kim et al., 2018 [158]	Mouse; C57BL/6 males; MPTP model.	saline (n = 10)/MPTP sham (n = 10)/MPTP + remote ischemic conditioning (leg) (n = 10)/MPTP + PBM (n = 10)/MPTP + remote ischemic conditioning + PBM (n = 10).	670 nm LED; Remote (dorsum); 50 mW/cm^2^ for 3 min; Pre-conditioned prior to MPTP injection.	Increased TH^+^ cells with remote ischemic conditioning and remote PBM; no additional benefit from combining treatments.
Ganeshan et al., 2019 [132]	Mouse; BABL/c males; MPTP model.	Saline (n = 10)/MPTP (n = 10)/MPTP + PBM 2 days pretreatment (n = 10)/ MPTP + PBM 5 days pre-treatment (n = 10)/MPTP + PBM 10 days pre-treatment (n = 10).	670 nm LED; Remote (dorsum + hind limbs); 50 mW/cm^2^ for 90 s for 2, 5 or 10 days prior to MTPT injection.	Decreased Fos^+^ in the caudate putamen complex for all pre-treatment groups; Increased TH^+^ cells and upregulated cell signalling, cell migration, oxidative stress response and blood–brain barrier modulation in 10-day pre-treatment group only.
Gordon et al., 2023 [143]	Mouse; C56BL/6 (head, abdomen and hind legs shaved). + Monkey (macaque); Adult male. All MPTP models.	Mouse: Control (n = 10)/MPTP (n = 10)/MPTP + PBM (head) (n = 10)/MPTP + PBM (abdomen) (n = 10)/MPTP + PBM (legs) (n = 10). Monkey: head (n = 1)/abdomen (n = 1)/hind legs (n = 1).	Mouse: 656 nm LED; Transcranial and remote (abdomen + hind legs); 50 mW/cm^2^ for 180 s; After MPTP injection, daily for 21 days. Monkey: 670 nm LED; Transcranial and remote (abdomen + lower legs); 50 mW/cm^2^ for 180 s; Immediately after and 4 h after MPTP injection over 5 days (10 treatments).	Mouse: No difference in mobility; Increased TH^+^ cells in SNc in all groups. Monkey (single individual): Lower clinical score for abdomen and leg treatment.
**WHOLE ANIMAL PBM**				
Vos et al., 2013 [151]	Drosophila; *pink1* null mutants; *park* null mutants; Rotenone toxin.	Variable numbers of mutant *pink1* flies/*pink1* controls/mutant *park* flies/*park* controls.	808 nm laser; Whole animal; 25 mW/cm^2^ for 100 s; Single dose.	Increased flight times; reduced mitochondrial defects and function; increased mitochondrial (complex IV) respiration.

PBM = photobiomodulation; LED = light emitting diode; MPTP = 1-methyl-4-phenyl-1,2,3,6-tetrahydropyridine.

Another solution to the penetration problem is the known systemic or abscopal effect of PBM [152,155,186], where PBM targeting an area of the body has benefits in other areas of the body. This is the so-called “middle man” suggested by Mitrofanis [182], who argues that the disease-modifying effect of transcranial PBM is either due to the light acting on other areas of the brain to compensate for the lack of dopamine or due to the systemic effect of PBM influencing the neurons of the SNc. Remote treatment has been shown to work well in animal models of Parkinson’s disease [143,158,161,187].

The proof-of-concept study by Liebert et al. [174] described above used both transcranial and abdominal PBM treatment, but with no ability to separate the effects of the two treatments. A second proof-of-concept study conducted by the same group [188] used only remote treatment (abdomen plus neck) on seven participants. The remote treatment alone produced similar results to the combination of treatments, with improvements in TUG tests, 10MWT, dynamic balance and MoCA seen after 12 weeks and one year of treatment. In addition, two participants with total anosmia improved to severe anosmia, using a validated smell test. This study demonstrated the potential of remote PBM as a treatment option for Parkinson’s disease.

The mechanism of the remote effect of PBM on Parkinson’s disease clinical signs and symptoms could, as discussed above, be a biochemical signal, circulating mitochondria, the enteric vagus nerve connection, or the microbiome–gut–brain axis. Very few studies have investigated the effect of PBM on the gut microbiome in animals or humans. The one study that has investigated gut microbiome changes in Parkinson’s disease patients with PBM treatment used a combination of abdominal and neck infrared lasers and transcranial and intranasal infrared LEDs [189]. While numbers in the study were small (12), the participants largely showed an improvement in their microbiome, with an improved Firmicutes–Bacteroidetes ratio, increased numbers in some anti-inflammatory SCFA-producing bacteria (such as *Bacteroides* and *Odoribacter)* and decreased numbers of pro-inflammatory potential pathogens (*Methanobrevibacter*, *Enterococcus*, *Eggerthella* and *Paraeggerthella*). This supports rodent model studies where PBM has been shown to increase the SCFA-producing bacterium *Allobaculum* in healthy mice [160], and to improve the rodent gut microbiome in models of Alzheimer’s disease [190], mild cognitive decline [191], bone regeneration [192] and type II diabetes [193]. Future clinical studies using remote PBM would benefit from analysis of gut microbiome changes.

**Table 2:** Summary of clinical trials using photobiomodulation to treat the symptoms of Parkinson’s disease.

Study	Participants	Design	PBM Parameters	Outcomes
**TRANSCRANIAL**				
Santos et al., 2019 [169]	Idiopathic Parkinson’s disease; H & Y stage 1–2; No severe dyskinesis; 20 males; 15 females.	RCT; Sham (low dose) (n = 18)/PBM (n = 17).	670 nm LED; Transcranial; 60 mW/cm^2^ for 60 s alternating between left and right temples with 30 s rest between six treatments; Twice per week for 9 weeks (18 treatments); “sham” received 5 s treatment.	Improved fast rhythm 10-metre walk test; No other significant improvements.
Hamilton et al., 2019 [170]	Idiopathic Parkinson’s disease.	Case studies of three patients; No placebo group.	home-made PBM devices; Transcranial. 670 nm + 850 nm or 810 nm LED strips; 30 min once per day OR 20 min twice per day.	A variety of self-reported improvements including motor (gait, tremor, fine motor skills and writing) and non-motor (sleeping, swallowing, sense of smell, speech, self-esteem and social interactions).
Hamilton et al., 2019 [171]	Idiopathic Parkinson’s disease.	Case studies of six patients (two repeated from previous report) [170]; No placebo group.	Variety of PBM devices; Transcranial + intranasal. Home-made device with 670 nm + 850 nm or 810 nm; 20 min or 30 min, once or twice per day OR Manufactured device with 670 nm + 810 nm LEDs; 20 min once or twice daily OR Intranasal device 660 nm; 20 min per day; Ongoing treatment up to 24 months.	A variety of self-reported improvements including motor (gait, tremor, rigidity, fine motor skills and writing) and non-motor (sleeping, swallowing, sweating, sense of smell, speech, self-esteem, social interactions and constipation).
Berman and Nichols 2019 [172]	NR	Case study; Two participants; No placebo group.	1068 nm LED; Transcranial; 2.6 mW/cm^2^ for 5 min; Twice daily for 28 days.	Improved clock drawing.
Tamae et al., 2020 [173]	Idiopathic Parkinson’s disease; Ages 30 to 80 years; H & Y stage 1.5–2; Muscle pain and stiffness.	Parallel group trial; Vacuum therapy (n = 6)/PBM (n = 6)/vacuum therapy + PBM (n = 6); No placebo group.	670 nm + 808 nm laser; Cervical to lumbar spine + dorsal trunk + forearms and palm; Power NR; time applied NR; Two PBM treatments per week for 3 weeks (six treatments).	Combined vacuum and PBM gave more pain relief (VAS) than either single treatment.
Liebert et al., 2019 [174]	Idiopathic Parkinson’s disease; Seven females; five males; Aged between 60 and 80 years.	Waitlist design; Immediate treatment (n = 6)/waitlist (n = 6); No placebo group.	904 nm laser; Abdominal; 30 mW/cm^2^ for 60 s on 9 points of abdomen + 1 point of neck; PLUS 810 nm LED; Transcranial; 100 mW/cm^2^ and 75 mW/cm^2^ for 20 min; PLUS 810 nm LED; Intranasal; 25 mW/cm^2^ for 25 min; All 3 times per week for 4 weeks, then 2 times per week for 4 weeks then 1 time per week for 4 weeks, then 3 times per week for up to 1 year.	Improvements in a range of motor (including gait, balance and fine motor skills) and cognitive outcome measures at both 12 weeks and 1 year; Measurable Hawthorne effect, with outcome measures further improved on treatment.
Bullock-Saxton et al., 2019 [176]	Idiopathic Parkinson’s disease.	RCT; Placebo (n = 10)/PBM (n = 10).	904 nm laser; Transcranial; 60 mW for 33 s; PLUS Intraoral; 60 mW for 33 s; Three PBM treatments per week for 4 weeks; 4 weeks no treatment; One PBM treatment per week for 4 weeks.	No significant changes but indications of positive effects for fine motor skills and dynamic balance.
Hong et al., 2021 [177]	Idiopathic Parkinson’s disease; 8 females; 12 males; Aged 30 to 80 years; H & Y stage 2–3.	Case series (n = 18); Combined therapy (PBM + h_2_ water); No placebo group.	940 nm LED; Neck; 6 mW/cm^2^ for 30 min for 5 consecutive days.	Improved UPDRS scores.
Liebert et al., 2022 [188]	Idiopathic Parkinson’s disease; Seven males; Aged between 60 and 80 years.	Case series (n = 7); No placebo group.	904 nm laser; Abdominal; 30 mW/cm^2^ for 120 s on 9 points of abdomen + 1 point of neck; For 12 weeks.	Improvements in a range of motor (including gait, balance and fine motor skills) and cognitive outcome measures at both 12 weeks and 1 year.
Bicknell et al., 2022 [189] Further results of Liebert et al.’s study [174]	Idiopathic Parkinson’s disease; Seven females; five males; Aged between 60 and 80 years.	Waitlist design; Immediate treatment (n = 6)/waitlist (n = 6); No placebo group.	904 nm laser; Abdominal; 30 mW/cm^2^ for 60 s on 9 points of abdomen + 1 point of neck; PLUS 810 nm LED; Transcranial; 100 mW/cm^2^ and 75 mW/cm^2^ for 20 min; PLUS 810 nm LED; Intranasal; 25 mW/cm^2^ for 25 min; All 3 times per week for 4 weeks, then 2 times per week for 4 weeks, then 1 time per week for 4 weeks, then 3 times per week for up to 1 year.	Changes to the microbiome following PBM.
Peci et al., 2023 [178]	Idiopathic Parkinson’s disease; 13 females; 25 males; Aged 60 to 75 years.	Parallel group trial; Physiotherapy (n = 26)/physiotherapy + PBM (n = 12); No placebo group.	810 nm LED array (256); Transcranial; 24 mW/cm^2^ for 18 min, twice per week for 4 weeks.	Combination of PBM and physiotherapy superior to physiotherapy alone for motor UPDRS scores.
Liebert et al., 2023 [175] 3-year follow-up of Liebert et al.’s 2019 study [174]	Idiopathic Parkinson’s disease; Seven females; five males; Aged between 60 and 80 years.	Waitlist design; PBM treatment (n = 6)/no treatment (n = 2); No placebo group.	904 nm laser; Abdominal; 30 mW/cm^2^ for 60 s on 9 points of abdomen + 1 point of neck; PLUS A variety of transcranial PBM devices.	Mobility and cognition outcomes continued to improve up to 2 years and did not decline at 3 years if PBM was maintained; no outcome measure declined to below pre-treatment (3 years previous) if PBM had continued.
Herkes et al., 2023 [180]	Idiopathic Parkinson’s disease; 20 females; 20 males; Aged 60 to 80 years.	Parallel randomised feasibility trial; Placebo (n = 20)/PBM (n = 20).	635 nm + 810 nm LED (20 + 20); Transcranial; 27 mW (635 nm) for 12 min then 52 mW (810 nm) for 12 min; Once per day, 6 days per week for 12 weeks; Placebo group switched to PBM after 12 weeks.	Safe and feasible treatment; No significant difference for MDS-UPDRS-III scores between groups at 12 weeks; Placebo group continued to improve after PBM treatment.
McGee et al., 2023 [181] Further results of Herkes et al.’s study [180]	Idiopathic Parkinson’s disease; 20 females; 20 males; Aged 60 to 80 years.	Parallel randomised feasibility trial; Placebo (n = 20)/PBM (n = 20).	635 nm + 810 nm LED (20 + 20); Transcranial; 27 mW (635 nm) for 12 min then 52 mW (810 nm) for 12 min; once per day, 6 days per week for 12 weeks; Placebo group switched to PBM after 12 weeks.	Responders to PBM treatment showed improvements in all sub-scores of MDS-UPDRS-III scores (facial expression, upper limb, lower limb, gait and tremors) after 12 weeks, while placebo responders showed improvements in only lower limb sub-score.

PBM = photobiomodulation; H&Y = Hoehn and Yahr; LED = light emitting diode; RCT = randomized placebo-controlled trial.

## 9. Conclusions

Unlike Parkinson’s disease medications with their significant and potentially serious side effects, PBM has been shown to have a remarkable safety record, both when applied to the periphery (remote application) and to the head. PBM also has many years of evidence to demonstrate a modulating effect on mitochondrial and inflammatory processes, and there is some suggestion that PBM may influence the microbiome. Since Parkinson’s disease involves mitochondrial dysfunction and neuroinflammation, with the involvement of the MGBA, treatment with PBM would have the potential to have some effect in this disease, as with other neurodegenerative diseases.

Evidence from pre-clinical models (in vitro and in vivo) suggests a promising role for PBM in the treatment of Parkinson’s disease. In animal models of Parkinson’s disease, PBM has been shown to provide neuroprotection to the neurons at risk from damage and death and to also reduce the clinical motor and behavioural signs. These effects occur whether the PBM is administered transcranially or remotely and whether the treatment is provided before, during or after the toxic event. Importantly, these effects, including neuroprotection, also occur in non-human primates. 

There is some evidence for the promise of PBM treatment for Parkinson’s disease. A number of small clinical trials suggest that participants can gain benefit from PBM treatment, with improvements in both the motor signs and non-motor symptoms of the disease, with transcranial and remote treatment. PBM is a very safe and non-invasive therapy that can be performed by the patients themselves. While larger, longer and appropriately powered and controlled trials are needed, it may be that PBM treatment may be one of the few, and perhaps only, treatments for Parkinson’s disease that can be translated from pre-clinical experiments to a clinical effect.

## Data Availability

No new data was generated in this study.
